# Blockade of Endothelin-1 Receptor Type B Ameliorates Glucose Intolerance and Insulin Resistance in a Mouse Model of Obstructive Sleep Apnea

**DOI:** 10.3389/fendo.2018.00280

**Published:** 2018-05-29

**Authors:** Jan Polak, Naresh M. Punjabi, Larissa A. Shimoda

**Affiliations:** ^1^Division of Pulmonary and Critical Care Medicine, Johns Hopkins University, Baltimore, MD, United States; ^2^Department for the Study of Obesity and Diabetes, Third Faculty of Medicine, Charles University, Prague, Czechia

**Keywords:** obstructive sleep apnea, endothelin, endothelin receptor, bosentan, diabetes

## Abstract

Obstructive sleep apnea (OSA) is associated with insulin resistance (IR) and glucose intolerance. Elevated endothelin-1 (ET-1) levels have been observed in OSA patients and in mice exposed to intermittent hypoxia (IH). We examined whether pharmacological blockade of type A and type B ET-1 receptors (ET_A_ and ET_B_) would ameliorate glucose intolerance and IR in mice exposed to IH. Subcutaneously implanted pumps delivered BQ-123 (ET_A_ antagonist; 200 nmol/kg/day), BQ-788 (ET_B_ antagonist; 200 nmol/kg/day) or vehicle (saline or propyleneglycol [PG]) for 14 days in C57BL6/J mice (10/group). During treatment, mice were exposed to IH (decreasing the FiO_2_ from 20.9% to 6%, 60/h) or intermittent air (IA). After IH or IA exposure, insulin (0.5 IU/kg) or glucose (1 mg/kg) was injected intraperitoneally and plasma glucose determined after injection and area under glucose curve (AUC) was calculated. Fourteen-day IH increased fasting glucose levels (122 ± 7 vs. 157 ± 8 mg/dL, PG: 118 ± 6 vs. 139 ± 8; both *p* < 0.05) and impaired glucose tolerance (AUC_glucose_: 19,249 ± 1105 vs. 29,124 ± 1444, PG AUC_glucose_: 18,066 ± 947 vs. 25,135 ± 797; both *p* < 0.05) in vehicle-treated animals. IH-induced impairments in glucose tolerance were partially ameliorated with BQ-788 treatment (AUC_glucose_: 21,969 ± 662; *p* < 0.05). Fourteen-day IH also induced IR (AUC_glucose_: 7185 ± 401 vs. 8699 ± 401; *p* < 0.05). Treatment with BQ-788 decreased IR under IA (AUC_glucose_: 5281 ± 401, *p* < 0.05) and reduced worsening of IR with IH (AUC_glucose_: 7302 ± 401, *p* < 0.05). There was no effect of BQ-123 on IH-induced impairments in glucose tolerance or IR. Our results suggest that ET-1 plays a role in IH-induced impairments in glucose homeostasis.

## Introduction

Obstructive sleep apnea (OSA) is a common condition characterized by repeated episodes of complete or partial upper airway collapse during sleep, resulting in repetitive drops in hemoglobin oxygen saturation and sleep fragmentation. Affecting about 5–15% of the general population (1, 2), OSA represents a considerable health burden, increasing the risk of hypertension (3, 4), cardiovascular disease (5–8) and all-cause mortality (9–11) independently of other traditional risk factors. Furthermore, research in the past decade suggests a role for OSA in the pathogenesis of insulin resistance (IR), glucose intolerance, metabolic syndrome and type 2 diabetes independent of age, obesity or fat distribution (12–14).

Although mechanisms linking OSA with impaired glucose homeostasis are not fully understood, several lines of evidence suggest that intermittent hypoxia (IH) plays a key role in inducing metabolic impairments. Experimental evidence from animal models suggests that both acute and prolonged exposure to IH worsened multiple aspects of glucose homeostasis including fasting glycemia, whole-body glucose tolerance and insulin sensitivity, muscle glucose uptake, hepatic glucose output, pancreatic β-cell function, viability and insulin secretion (15–21). Furthermore, impaired insulin sensitivity and pancreatic dysfunction were observed in healthy human volunteers acutely exposed to IH (22). It has been suggested previously that modulation of autonomic activity, altered corticotropic function, increased oxidative stress, activation of inflammatory pathways or higher circulating adipokines might represent the intermediate molecular and endocrine mechanisms imposing the metabolic toll of intermittent hypoxemia (23, 24). More recently, elevated levels of a circulating hormone, endothelin-1 (ET-1), and increased expression of cellular ET-1 receptors were demonstrated after IH exposure *in vitro* and in patients with OSA (25–31). Elevated ET-1 levels play a key role in abnormal carotid body functioning, leading to augmented sympathetic activation and development of systemic hypertension found in OSA (32, 33), while continuous positive airway pressure therapy effectively lowered blood pressure as well as ET-1 levels (27).

Substantial evidence suggests a potential link between ET-1 and the pathogenesis of metabolic impairments associated with type 2 diabetes. For example, ET-1 levels are elevated in patients with type 2 diabetes and metabolic syndrome (34, 35) as well as in animal models of diabetes (36, 37). Furthermore, ET-1 can directly interfere with cellular insulin signaling (38–42), enhance hepatic glucose output (43), diminish glucose uptake (40, 44–47), impair insulin secretion (48) and stimulate lipolysis in adipose tissue (39, 49) – effects directly contributing to hyperglycemia and development of type 2 diabetes. A causal role for ET-1 is further corroborated by studies documenting improved glucose homeostasis and insulin sensitivity after ET-1 receptor blockade in insulin-resistant rodents, healthy volunteers and insulin-resistant subjects (47, 50–52).

Collectively, these findings indicate that chronically elevated ET-1 levels could play a fundamental role in the development of IR and glucose intolerance; however, it remains unclear whether ET-1 is involved in the development of metabolic impairments associated with IH and OSA and whether pharmacological treatment with ET-1 receptor antagonists would provide metabolic benefit in these subjects. In this study, we employed a mouse model of OSA to investigate whether selective pharmacological blockade of ET-1 type A (ET_A_) or type B (ET_B_) receptors would ameliorate IH-induced alterations in glucose metabolism such as whole-body glucose tolerance, insulin sensitivity and insulin secretion.

## Materials and Methods

All procedures involving animals were approved by the Johns Hopkins University Animal Care and Use Committee and followed NIH principles of laboratory animal care.

### Selective ET-1 Receptor Antagonist Treatment

Adult male C57BL6/J mice (Jackson Labs, age 6–8 weeks) were used for this study. All drugs and vehicles were administered *via* osmotic minipumps (Model 1002, Alzet, Cupertino, CA, USA) implanted subcutaneously in the dorsal region under isoflurane anesthesia (1–2% isoflurane in oxygen, Baxter, Deerfield, IL, USA) and incisions closed with 2–3 sutures. Pumps were filled with 100 µl of: (a) BQ-123 (0.407 mg/mL dissolved in saline; American Peptide, Sunnyvale, CA, USA), a selective ET_A_ antagonist; (b) BQ-788 (0.509 mg/mL dissolved in propylene glycol; American Peptide, Sunnyvale, CA, USA), a selective ET_B_ antagonist; (c) saline (vehicle for BQ-123) or (d) propylene glycol (vehicle for BQ-788; Sigma-Aldrich, St. Louis, MO, USA). The osmotic minipumps delivered a constant flow-rate of 0.25 μl/h, resulting in a dose of 200 nmol/kg/day of BQ-123 or BQ-788 in each animal (~25 g body weight).

### Protocol for IH

All animals were allowed a recovery period of 24 h after surgery before subsequent exposures. Mice were housed in custom-modified cages connected with plastic tubing to a gas control delivery system regulating the flow of nitrogen, oxygen and compressed air into cages as previously described (19, 53). IH was induced by programmable solenoid valves and flow regulators, which were used to alter the composition of the gas within the cages so that during each cycle of hypoxia, the inspired fraction of oxygen (FiO_2_) was reduced from 21% to 6–7% over a period of 30 s and rapidly returned to 21% during the following 30 s. For the control exposure (intermittent air [IA]), animals were exposed to alternating periods of air (FiO_2_ = 21%) simulating a pattern of air flow at intervals identical to intermittent hypoxic exposure. On average, 60 episodes of oxygen desaturation were induced per hour mimicking oxyhemoglobin desaturations observed in severe OSA (53). All animals were housed at room temperature and subjected to a 12 h light/dark cycle. Exposures to IH and control exposures were conducted for 12 h during the dark phase of the light cycle. At the end of exposures, plasma samples were obtained or metabolic functions were assessed, as described below. Blood pressure was measured in acclimatized and trained animals (three exposures to the measuring routine on separate days before recordings were performed) using the non-invasive tail-cuff technique (CODA system, Kent Scientific, Torrington, CT, USA). Because IH can induce some weight loss, food intake was recorded and adjusted daily such that the body weight of groups followed the same trajectory. Animals were sacrificed by isoflurane overdose on the last day of the exposures.

### Intraperitoneal Glucose Tolerance Test (GTT) and Insulin Tolerance Test (ITT)

An intraperitoneal GTT was performed after a 5 h fast (*n* = 10 for each group). Following a glucose injection (1 g/kg glucose dissolved in saline), glucose levels were measured at 0, 10, 20, 30, 60, 90 and 120 min after the glucose injection from tail-snip blood samples using a glucometer (Accu-Check Aviva, Roche, Indianapolis, IN, USA). An ITT (*n* = 10 per group) was similarly performed after a 2 h fast by measuring blood glucose levels at 0, 10, 20, 30, 40, 50, 60, 90 and 120 min after injecting 0.5 IU/kg of insulin (Humulin R, 100 U/mL, Eli Lilly, Indianapolis, IN, USA). The GTT and ITT measurements were performed in a distinct set of freely moving animals with continued IH or control exposures. Glucose-induced insulin secretion (*n* = 10 per group) was assessed by measuring insulin levels using the Mouse Ultrasensitive EIA kit (Alpco, Salem, NH, USA) in plasma separated from blood obtained by cardiac puncture (after quick isoflurane anesthesia) 30 min after a 1 g/kg glucose injection. Fasting insulin levels were evaluated in separate group of animals. Blood was collected in tubes containing heparin (5 µL heparin/1 mL blood), centrifuged at 2000 g for 10 min to separate plasma from circulating blood cells, plasma transferred to a clean tube and samples frozen immediately and kept at −80°C until analysis. The homeostasis model assessment (HOMA) was used to derive HOMA-β ([20·Insulin]/[Glucose-3.5]) as a measure of β-cell function (54). Plasma ET-1 levels were measured in identical plasma samples as fasting insulin levels using the Endothelin-1 Quantikine ELISA Kit (R&D Systems Inc., Minneapolis, MN, USA).

### Statistical Analysis

Data are reported as mean and standard error of the mean (SEM). The effect of ET-1 receptor blockade was assessed by comparing the GTT- and ITT-derived parameters between the IH (drug- and vehicle-treated conditions) and their respective control exposure groups. Differences between groups in ITT and GTT glucose profiles were examined using ANOVA with repeated measures while between-group differences in all other variables were tested using two-way ANOVA with exposure group (IH versus control exposure) and treatment group (antagonist versus vehicle) as the between-subjects factors. Area under glucose curve (AUC) during the ITT was calculated using the trapezoidal rule using the entire testing period of 120 min. Body weight was used as a covariate in ANOVA. All tests were performed in SPSS for Windows 13.0 (SPSS Inc., Chicago, IL, USA). A *p* value <0.05 was considered statistically significant.

## Results

### Cardiovascular Parameters

In saline-treated animals, exposure to IH increased both systolic and diastolic systemic blood pressure (Table 1). Additionally, IH exposure increased heart rate in the saline-treated group (557 ± 6 vs. 654 ± 77 beats/min, *p* < 0.05). In animals under control exposures (IA), none of the treatments (BQ-123, propylene glycol or BQ-788) had a significant effect on blood pressure or heart rate, but in all three groups, the IH-induced increase in blood pressure was prevented (ANOVA, *p* < 0.05). The IH-induced increase in heart rate was not ameliorated by BQ-123 treatment (599 ± 21 vs. 682 ± 20 beats/min, *p* < 0.05). In animals treated with propylene glycol or BQ-788, heart rate increased with IH by 15% and 5%, respectively, although in both cases, the increase in heart rate was not statistically significant.

**Table 1:** Biochemical and physiological variables in all treatment groups.

	Saline		BQ123		Propylene glycol		BQ788	
	Control	IH	Control	IH	Control	IH	Control	IH
BP_systolic_ (mmHg)	149 ± 8	185 ± 6*	155 ± 6	148 ± 8	152 ± 4	145 ± 5	134 ± 4	147 ± 4
BP_diastolic_ (mmHg)	123 ± 7	151 ± 5*	122 ± 6	108 ± 6	113 ± 2	115 ± 7	96 ± 3	110 ± 5
Heart rate (bpm)	557 ± 6	654 ± 77	599 ± 21	682 ± 20	631 ± 46	724 ± 37	701 ± 44	735 ± 23
Glucose (mg/dL)	122 ± 7	157 ± 8*	138 ± 10	171 ± 8*	118 ± 6	139 ± 8*	119 ± 8	137 ± 6*
Insulin (ng/mL)	0.15 ± 0.03	0.21 ± 0.05	0.33 ± 0.07^‡^	0.26 ± 0.04	0.17 ± 0.08	0.20 ± 0.02	0.27 ± 0.06^‡^	0.14 ± 0.01*^,^^‡^
ET-1 (pg/mL)	1.50 ± 0.29	1.5 ± 0.21	2.0 ± 0.32	1.81 ± 0.21	1.23 ± 0.11	1.35 ± 0.16	1.06 ± 0.07^‡^	1.38 ± 0.15*
HOMA-β	18.9 ± 2.6	16.3 ± 3.9	32.2 ± 8.1^‡^	26.7 ± 3.0^‡^	23.2 ± 9.3	11.2 ± 2.9*	24.0 ± 5.0	12.4 ± 1.5*
Peak insulin (ng/mL)	0.21 ± 0.01	0.37 ± 0.06*	0.65 ± 0.15^‡^	0.31 ± 0.02	0.24 ± 0.12	0.28 ± 0.02	0.4 ± 0.14	0.27 ± 0.03
HOMA-β peak	13.7 ± 1.4	12.9 ± 2.0	42.2 ± 9.6^‡^	13.2 ± 1.6*	19.0 ± 9.0	16.3 ± 2.0	25.5 ± 9.0	17.4 ± 1.7
Body weight (g)	24.0 ± 0.4	25.3 ± 0.4	23.9 ± 0.3	25.2 ± 0.3	24.6 ± 0.3	24.8 ± 0.3	24.8 ± 0.3	24.5 ± 0.3

*p < 0.05 when compared to a control condition (*t*-test).

*^‡^*p* < 0.05 when compared to vehicle treatment (*t*-test)*.

### ET-1 Levels

The basal ET-1 level in mice under control exposure and receiving saline was 1.5 ± 0.29 pg/mL. Surprisingly, exposure to IH had no effect on plasma ET-1 levels in saline-treated mice (Table 1). Similarly, there was no significant difference in basal ET-1 levels in mice under control exposures receiving propylene glycol, nor were ET-1 levels changed by IH in propylene glycol-treated mice. Under control exposures, treatment with BQ-123 increased ET-1 levels by 30%, although this increase did not quite reach statistical significance (*p* = 0.09). In contrast, administration of BQ-788 had the opposite effect and diminished ET-1 levels by 14% (*p* < 0.05) in mice under control exposures. With exposure to IH, ET-1 plasma levels increased in mice receiving BQ-788 by 30% (*p* < 0.05), but were not significantly altered in IH mice that received BQ-123.

### Glucose Tolerance

**Figure 1:**
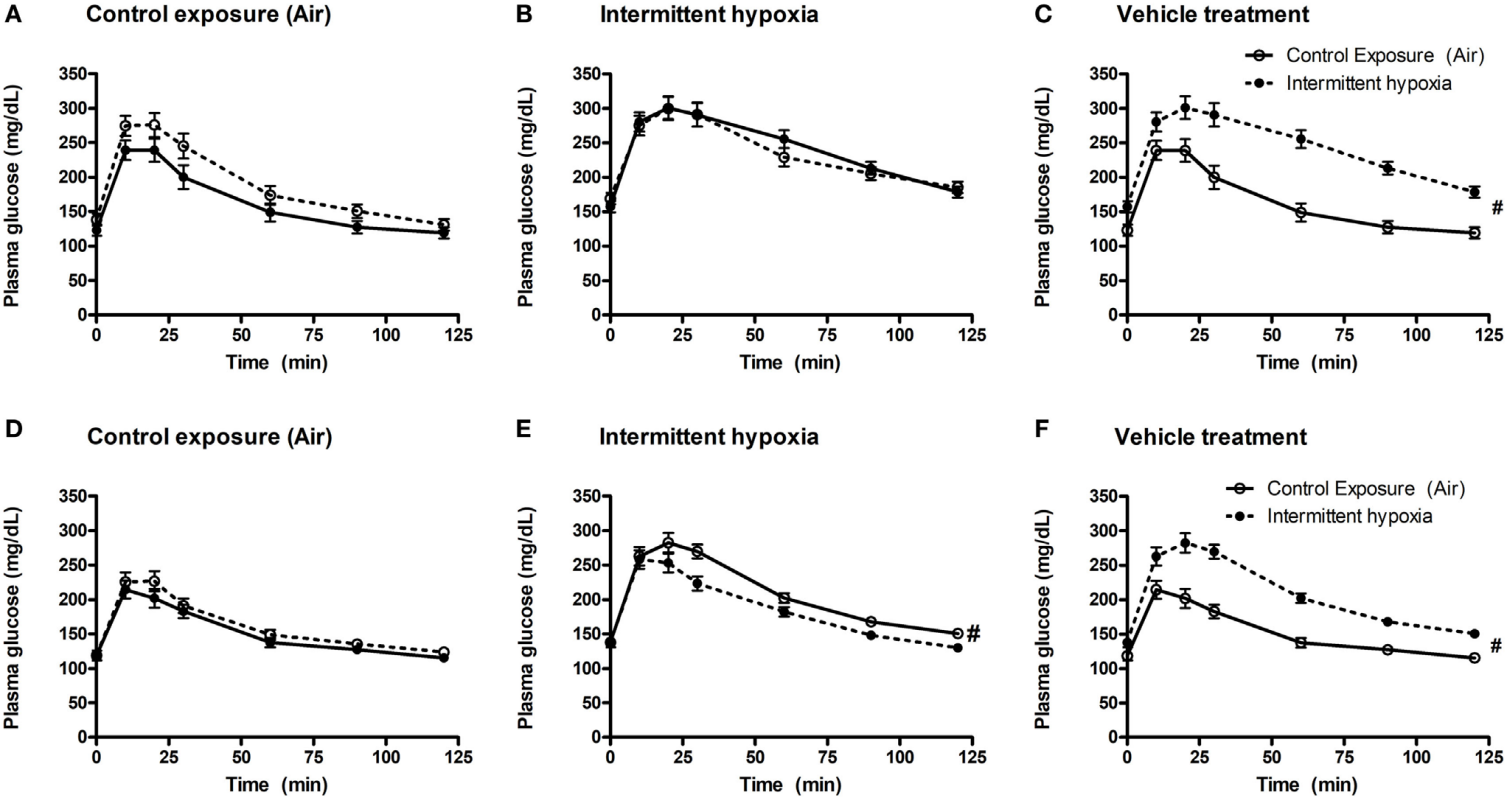
Intraperitoneal glucose tolerance tests (GTTs) in BQ-123 **(A,B)**, BQ-788 **(D,E)** and vehicle **(C,F)** treated groups. Data were analyzed using repeated measures ANOVA analysis of the GTT profiles investigating interaction between exposure groups (drug-treated versus vehicle-treated) and time of GTT. Upper panels: BQ-123 and saline treated groups. Lower panels: BQ-788 and propylethylene glycol (vehicle). ^#^ indicates *p* < 0.05 for differences between drug-treated and vehicle-treated groups. *N* = 10 for each group.

**Figure 2:**
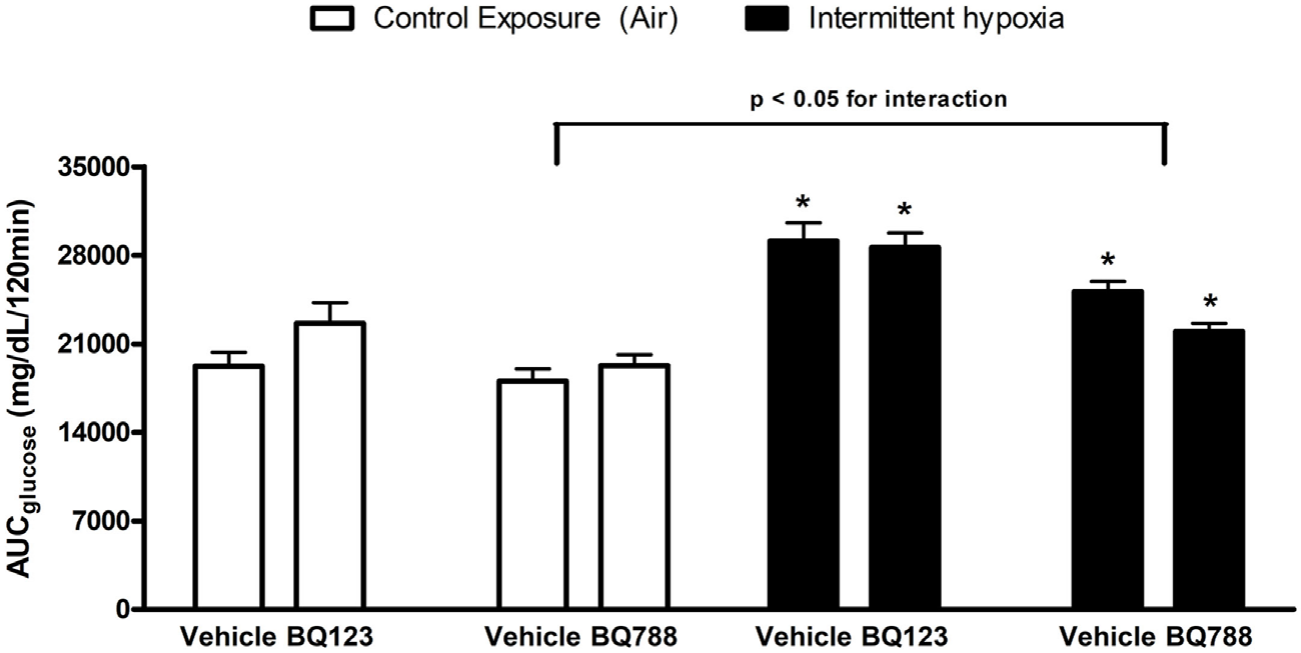
Area under glucose curve (AUC) during intraperitoneal glucose tolerance test. Interaction between hypoxic exposure and drug treatment was investigated using two-way ANOVA. * indicates *p* < 0.05 for differences between a control group (intermittent air) and a group exposed to intermittent hypoxia (IH). *N* = 10 for each group.

In vehicle-treated animals, a 2-week exposure to IH elevated fasting glycemia by 29% (saline) and 18% (propylene-glycol) and impaired glucose tolerance as assessed by GTT (Figure 1) and area under the glucose curve (AUC_glucose_; Figure 2). Neither BQ-123 nor BQ-788 altered fasting glycemia under control exposures and neither prevented the IH-induced increase in fasting glycemia (Table 1). Importantly, in animals exposed to IH, only treatment with BQ-788 significantly improved glucose tolerance, limiting worsening of glucose tolerance with IH to 12%, compared to a 28% change in vehicle-treated mice (Figures 1A,B) or a 21% change in mice treated with BQ-123 (Figures 1C,D). There was also a significant interaction between treatment with BQ-788 (versus vehicle) and exposure to IH (ANOVA, *p* < 0.05 for interaction).

### Insulin Levels and β-Cell Function

In saline-treated animals, with exposure to IH there was a trend for higher fasting blood insulin levels, although the change did not reach statistical significance (*p* = 0.34; Table 1). In animals under control exposures, propylene glycol had no effect on fasting insulin, whereas both ET-1 receptor antagonists increased fasting insulin levels, by 113% for BQ-123 and by 56% for BQ-788 (both *p* < 0.05). In propylene glycol-treated mice exposed to IH, fasting insulin levels were also slightly higher, but the difference did not reach statistical significance (*p* = 0.82). In both BQ-123- and BQ-788-treated mice exposed to IH, insulin levels were reduced compared to control exposures, although this reduction was only statistically significant for mice treated with BQ-788. However, no interaction was observed between exposure to IH and ET-1 receptor antagonist treatments, suggesting that neither BQ-123 nor BQ-788 modified the effects of IH on fasting insulin (ANOVA, *p* > 0.05).

Assessment of the HOMA-β index revealed that in saline-treated mice, exposure to IH had no effect on pancreatic β-cell function (Table 1). In mice receiving propylene glycol, the HOMA-β index under control exposure was not different from saline-treated mice; however, the HOMA-β index significantly decreased with IH. Treatment with BQ-123 improved insulin secretion under control exposure and IH conditions (both *p* < 0.05). In contrast, BQ-788 had no significant effect on HOMA-β index under control exposures, but the HOMA-β index was reduced in mice receiving BQ-788 during IH. No interaction was observed between exposure to IH and ET-1 receptor antagonist treatments, suggesting that neither BQ-123 nor BQ-788 modified the effects of IH on β-cell function.

### Insulin Sensitivity and Body Weight Trajectory

**Figure 3:**
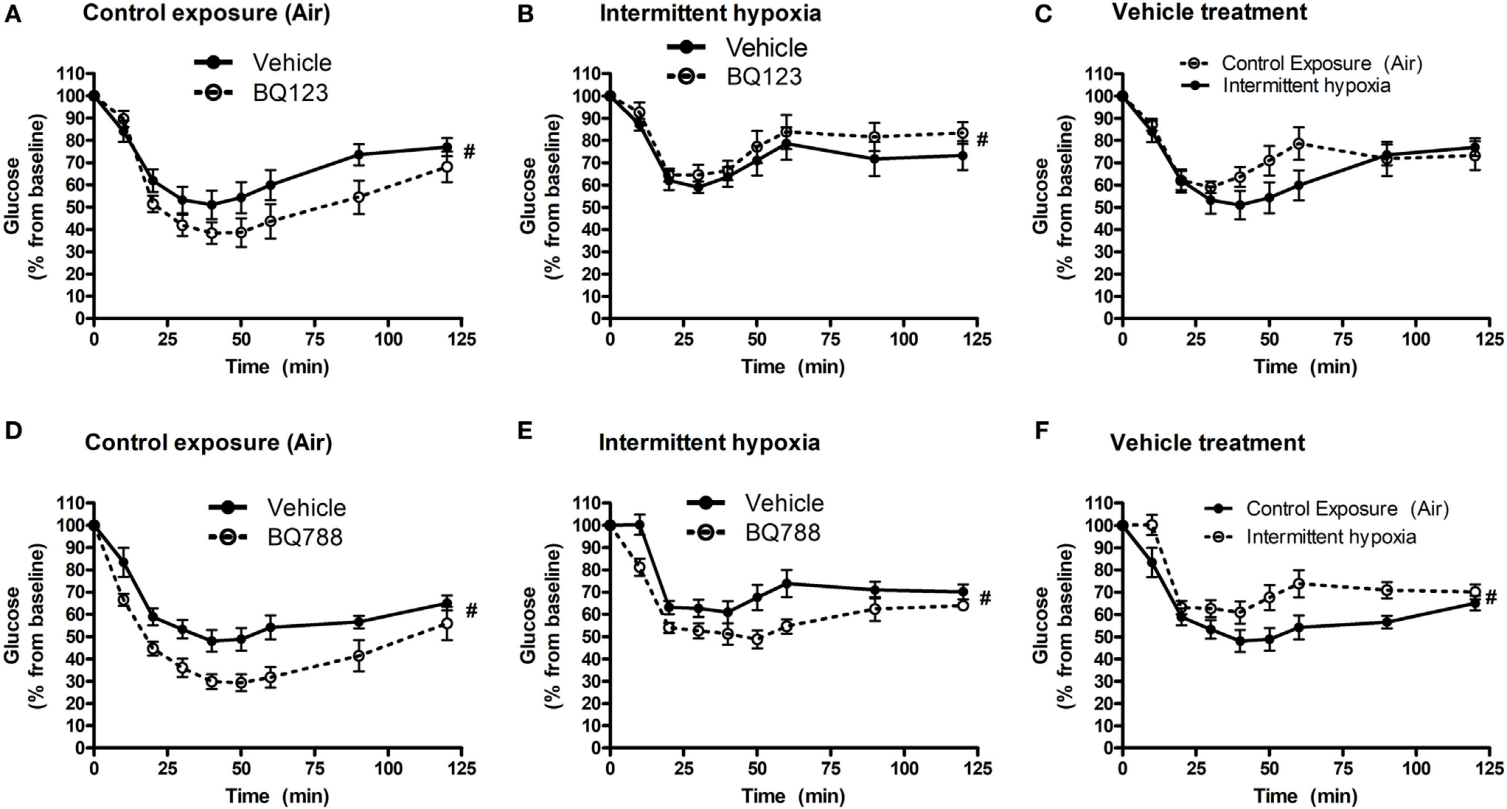
Intraperitoneal insulin tolerance tests (ITTs) in BQ-123 **(A,B)** and BQ-788 **(D,E)** and vehicle **(C,F)** treated groups. Data were analyzed using repeated measures ANOVA analysis of the glucose tolerance test (GTT) profiles investigating interaction between exposure groups (drug-treated versus vehicle-treated) and time of GTT. Upper panels: BQ-123- and saline (vehicle)-treated groups. Lower panels: BQ-788- and propylene glycol (vehicle)-treated groups ^#^ indicates *p* < 0.05 for differences between drug-treated versus vehicle-treated groups. *N* = 10 for each group.

**Figure 4:**
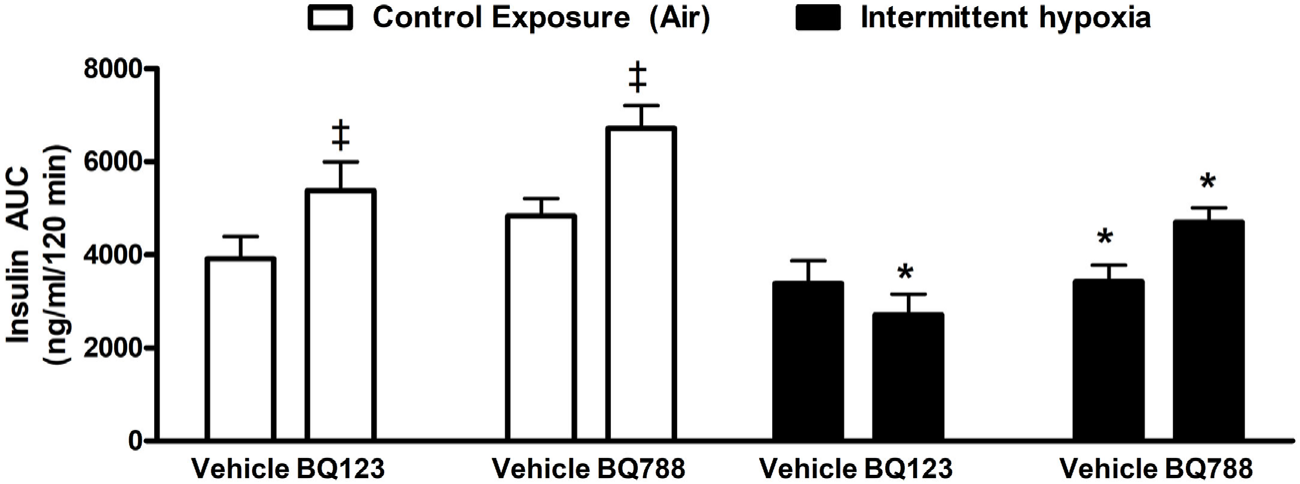
Area under glucose curve (AUC) during intraperitoneal insulin tolerance test. Interaction between hypoxic exposure and drug treatment was investigated using two-way ANOVA. * indicates *p* < 0.05 for differences between a control group (intermittent air) and a group exposed to intermittent hypoxia.^‡^ indicates *p* < 0.05 for differences between a drug-treated versus vehicle-treated groups. *N* = 10 for each group.

**Figure 5:**
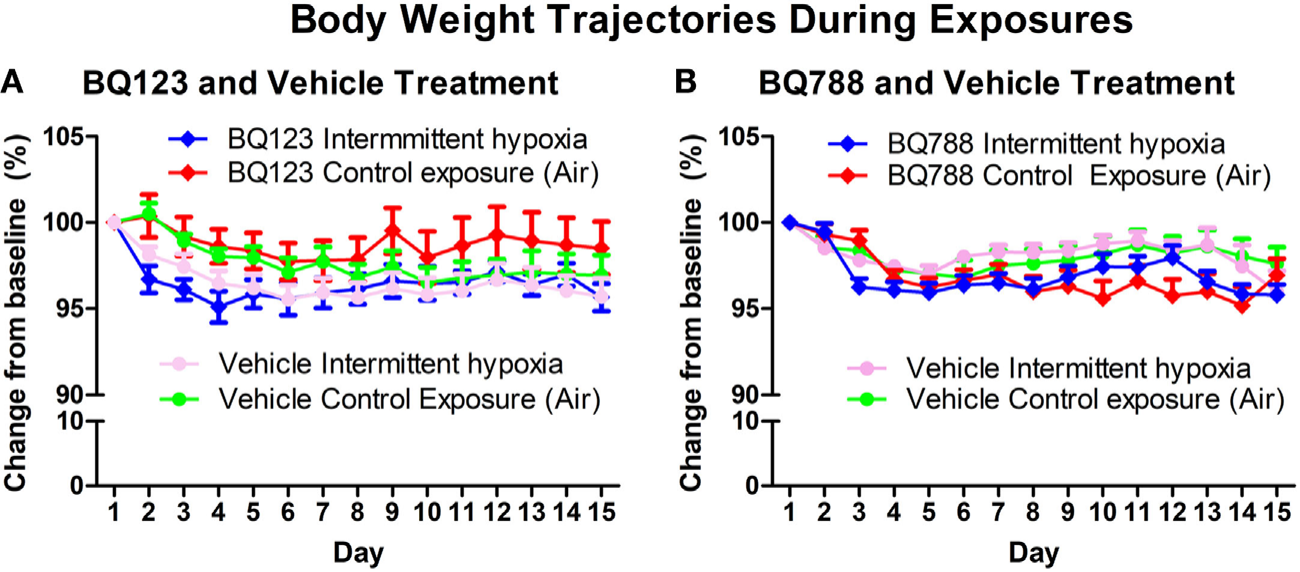
Body weight trajectories of vehicle and BQ-123 **(A)** and BQ-788 **(B)** treated groups during control exposures and intermittent hypoxia exposure. There were no differences in body weight trajectories between groups (two-way ANOVA). *N* = 15 for each group.

Analysis of individual glucose profiles and AUC_glucose_ revealed that exposure to IH worsened insulin sensitivity across all groups (Figures 3 and 4). While propylene glycol had no significant effect on insulin sensitivity, treatment with either BQ-123 (Figures 3A,B) or BQ-788 (Figures 3C,D) improved insulin sensitivity under control conditions (*p* < 0.05; Figure 4). Only BQ-788 administration also improved insulin sensitivity under IH conditions (*p* < 0.05). As a result, insulin sensitivity of mice exposed to IH while treated with BQ-788 was nearly identical to insulin sensitivity of vehicle-treated mice under control exposures (*p* = 0.76). Exposure to IH induced initial weight loss (~3–5%, *p* < 0.05); however, there were no differences between control exposures and IH groups due to pair-feeding. Body weight trajectories of all exposure groups during the experiment are shown in Figure 5.

## Discussion

In the present study, we investigated whether pharmacological inhibition of ET-1 signaling with the selective ET_A_ or ET_B_ receptor antagonists, BQ-123 or BQ-788, respectively, would ameliorate the detrimental impact of IH on glucose homeostasis. We showed that treatment with BQ-788, but not BQ-123, diminished IH-induced glucose intolerance. Additionally, both antagonists improved insulin sensitivity under control conditions; however, only BQ-788 remained effective during IH. These results suggest that ET-1 signaling *via* the ET_B_ receptor contributes to IH-induced disturbances in glucose homeostasis.

In the current study, exposure to IH elevated fasting glycemia, impaired glucose tolerance and induced IR. These findings are consistent with previous results from our lab and others using acute and prolonged IH exposure in obese and non-obese animals (15–17, 19, 21, 55, 56). Targeting ET-1 signaling as a therapy for metabolic impairments associated with OSA was motivated by numerous studies demonstrating elevated ET-1 plasma levels in humans or animals with type 2 diabetes (34–37, 57), as well as by reports suggesting direct involvement of ET-1 signaling in the pathogenesis of glucose intolerance and hyperglycemia (18, 38, 40–49). Our study was also motivated by reports of elevated ET-1 plasma levels in patients with OSA (25–27, 29–31). Similar to other groups (58, 59), we employed continuous subcutaneous administration of selective receptor antagonists to inhibit ET-1 signaling. Treatment with ET_A_ receptor antagonists under control conditions slightly increased fasting glucose and insulin levels as well as worsened glucose tolerance, although none of these changes reached statistical significance. We attribute these observations to IR-inducing effects of ET-1, as plasma ET-1 levels increased by 33% after BQ-123 treatment. Surprisingly, ET_A_ receptor antagonist treatment provided no beneficial metabolic effects under exposure to IH.

The insulin-sensitizing effects of BQ-123 as well as BQ-788 under control exposures suggest that ET-1 is involved in the regulation of whole-body IR, exerting an overall IR-promoting effect, which is alleviated after ET_A_ or ET_B_ receptor blockade, congruent with a previous study (42). Although speculative, we suggest that improvements in insulin sensitivity after ET_B_ blockage are driven by the effects on adipose tissue where ET-1 induces IR *via* ET_B_ receptors (60), while the beneficial effect of ET_A_ blockade might be mediated by ET_A_ receptors *via* the reduced vasoconstrictor tone and enhanced microvascular capillary bed recruitment after insulin injection and thus faster glucose removal from circulation (61, 62). In contrast, treatment with ET-1 receptor antagonists had no effect on fasting glucose levels neither under control exposures nor in response to IH exposure, suggesting that ET-1 is probably not a major determinant of hepatic glucose output *in vivo* as also supported by studies showing no effect of bosentan, a non-selective ET_A_/ET_B_ receptor antagonist, on fasting glucose levels in diabetic rats (61) and by the fact that expression of ET-1 receptors was not detected in rat hepatocytes (63).

Interestingly, only ET_B_ receptor antagonists improved insulin sensitivity during IH exposure and ameliorated the IH-induced impairment in glucose tolerance, suggesting that ET_B_ receptor activation and signaling might be a causal factor in driving development of adverse metabolic impairments associated with IH exposure. As a previous study demonstrated that ET_B_, but not ET_A_, receptor signaling induced IR in visceral human adipocytes (60), we speculate that IR is induced by intermittent hypoxic exposure particularly in adipose tissue *via* enhanced ET_B_ receptor expression or augmentation of intracellular signaling. Importantly, adipose tissue IR can be induced by intermittent as well as sustained hypoxic exposure (64–66). Treatment with ET_B_ receptor antagonist targets this dysregulation and leads to improved insulin sensitivity and, subsequently, to lower plasma glucose levels in intraperitoneal GTT as we observed. Additionally, enhanced muscle glucose uptake and increased insulin sensitivity was reported in healthy volunteers, insulin resistant subjects and *in vitro* models after ET_B_ receptor blockade (40, 47, 50). Besides direct effects on insulin signaling/sensitivity, muscle glucose uptake can also be increased by an increase in nutritive capillary bed blood flow (61, 62). As higher ET-1 levels mediate ET_B_ receptor-dependent vasoconstriction (67, 68), it is plausible that ET_B_ receptor blockade leads to vasodilation at least in some vascular beds (69) and might thus contribute to enhanced glucose uptake from circulation. The focus on IR as a major target of BQ-788 effects is further supported by the fact that parameters of β-cell function remained unaffected by ET_B_ receptor antagonist. Further studies are needed to better elucidate opposite metabolic effects of ET_A_ and ET_B_ receptor signaling in metabolically relevant tissues in the context of glucose homeostasis (49, 60).

In contrast to ET_B_ receptor blockade, administration of an ET_A_ receptor antagonist had no impact on IH-induced alterations in glucose homeostasis probably due to elevation of plasma ET-1 levels after BQ-123 administration observed in our study and reported also by others (51, 70–74). Additionally, elevated glucose levels (e.g., during a postprandial state) can increase ET-1 production (75–77). It is thus plausible to hypothesize that elevated ET-1 levels in BQ-123 treated mice augmented signaling through unblocked ET_B_ receptors with adverse metabolic outcomes. Finally, the lack of BQ-123 metabolic effect might be caused by insufficient dosing; however, this explanation seems less likely as the development of arterial hypertension typically associated with IH exposure (58) was prevented by BQ-123 administration in our study.

In the present study, we were unable to detect a significant elevation of plasma ET-1 with intermittent hypoxic exposure which is in contrast to reports documenting that exposure to sustained hypoxia or IH acutely increased plasma ET-1 levels (32, 78–80). However, it was also demonstrated that despite continued exposure to hypoxia, plasma ET-1 levels gradually fall and eventually return to pre-exposure levels in 14 days (80). It is also possible that the clearance of ET-1 from circulation was increased possibly through the increase in the number of endothelial ET_B_ receptors. Finally, it is important to note that plasma ET-1 levels are subjected to rapid clearance, particularly in lungs, and thus do not necessarily reflect tissue ET-1 concentrations.

The current study has several important limitations that merit discussion. First, the duration of exposure and antagonist treatment was limited to 2 weeks due to the limited size/volume of implantable osmotic minipumps. While we and other groups have shown that a 2-week exposure is sufficient to induce a diabetic phenotype in mice (17, 19, 81), longer exposures and extension of this work to obese and/or older animals will provide important knowledge on metabolic consequences of chronic IH exposure under conditions more closely mimicking those observed in OSA patients. Second, GTT and ITT provide important information regarding whole-body glucose and insulin sensitivity, but more specific investigations will be necessary to separate the effects of IH and antagonist treatment on specific tissue processes such as insulin secretion, glucose uptake in muscle or hepatic glucose output. Third, it is important to note that several metabolically related parameters, e.g., adiposity, spontaneous food intake and physical activity were not measured in this study leaving the impact of intermittent hypoxic exposure and drug treatment on these variables unknown. It should be noted that exposure to IH induces weight loss (64), which led authors of this study as well as other investigators to weight-match control and IH groups throughout the exposures by monitoring food available to both groups to prevent differences in body weight trajectories. These differences, together with variations in duration, severity and type of hypoxic exposures (sustained versus intermittent), need to be considered in interpretation of the metabolic outcomes between studies. Finally, we used C57BL6/J mice that are susceptible to the development of diabetes due to a mutation in the nicotinamide nucleotide transhydrogenase gene (82). Future research will be required to determine whether the results obtained in this model using ET_B_ receptor antagonism translate into management of type 2 diabetes in OSA patients.

In conclusion, we found that administration of BQ-788 reduced the metabolic toll of IH exposure in mice, improving insulin sensitivity and limiting the development of glucose intolerance. These results suggest that the adverse metabolic consequences observed under IH are at least partially mediated by ET-1 acting specifically through its ET_B_ receptor, possibly *via* an effect on muscle insulin sensitivity. While it is clear that these initial studies are just a beginning, our work provides a possible rationale for exploring the potential of ET_B_ receptor antagonism as a pharmacological treatment of metabolic abnormalities associated with OSA.

## Ethics Statement

All procedures involving animals were approved by the Johns Hopkins University Animal Care and Use Committee and followed NIH principles of laboratory animal care.

## Author Contributions

JP participated in study design, conducted experiments, performed biochemical analysis, analyzed data and prepared a manuscript. LS and NP developed study design, supervised experiments, conducted experiments and participated in manuscript preparation.

## Conflict of Interest Statement

The authors declare that the research was conducted in the absence of any commercial or financial relationships that could be construed as a potential conflict of interest.

## Supplementary Material

Figure S1 Estimated food intake during exposures. The amount of food provided daily per cage and food left the next day per cage was recorded. No differences were observed between groups, however this does not represent the true food intake of individual animals (some food is lost in bedding during chowing, etc…).
