# Supplementary figures and images for: Blockade of Endothelin-1 Receptor Type B Ameliorates Glucose Intolerance and Insulin Resistance in a Mouse Model of Obstructive Sleep Apnea
Source: Front Endocrinol (Lausanne). 2018 May 29;9:280. doi: 10.3389/fendo.2018.00280 (PMC5986958; doi:10.3389/fendo.2018.00280)

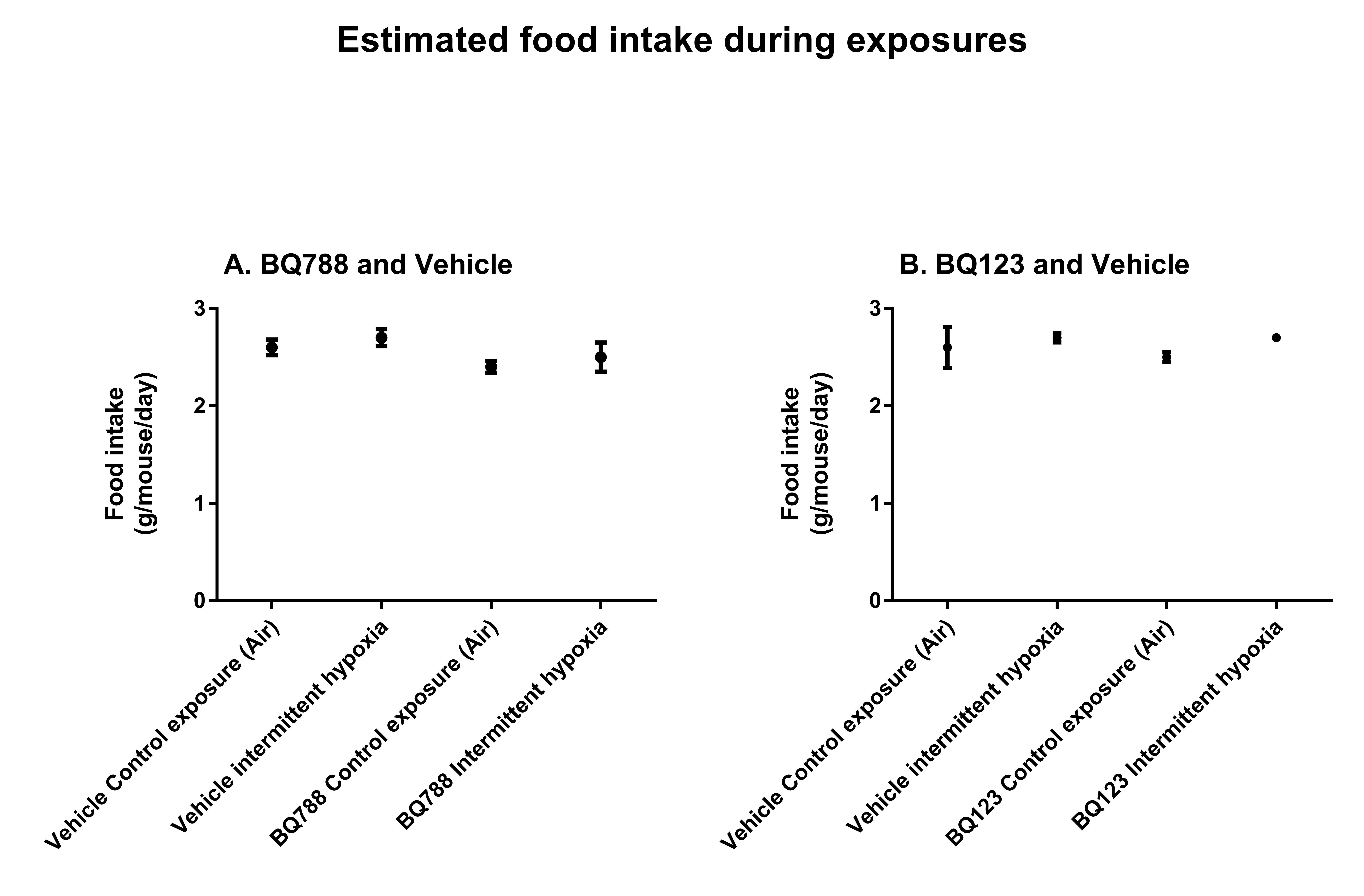

Supplement: Figure S1 — Estimated food intake during exposures. The amount of food provided daily per cage and food left the next day per cage was recorded. No differences were observed between groups, however this does not represent the true food intake of individual animals (some food is lost in bedding during chowing, etc…). [file image_1.jpg]
